# The development and validation of measures to assess cooking skills and food skills

**DOI:** 10.1186/s12966-017-0575-y

**Published:** 2017-09-02

**Authors:** Fiona Lavelle, Laura McGowan, Lynsey Hollywood, Dawn Surgenor, Amanda McCloat, Elaine Mooney, Martin Caraher, Monique Raats, Moira Dean

**Affiliations:** 1 Institute for Global Food Security, School of Biological Sciences, Queen’s University Belfast, Belfast, BT9 5AG UK; 2 Centre for Public Health, School of Medicine, Dentistry and Biomedical Sciences, Queen’s University Belfast, Belfast, UK; 3 Department of Hospitality and Tourism Management, Ulster University Business School, Ulster University, Coleraine, UK; 4 Department of Home Economics, St. Angela’s College, Sligo, Ireland; 5 Centre for Food Policy, Department of Sociology, School of Arts and Social Sciences, City University London, London, UK; 6 Food, Consumer Behaviour and Health Research Centre, School of Psychology, University of Surrey, England, UK

**Keywords:** Cooking skills, Food skills, Development, Validation, Cross-sectional, Intervention, Measure, Obesity

## Abstract

**Background:**

With the increase use of convenience food and eating outside the home environment being linked to the obesity epidemic, the need to assess and monitor individuals cooking and food skills is key to help intervene where necessary to promote the usage of these skills. Therefore, this research aimed to develop and validate a measure for cooking skills and one for food skills, that are clearly described, relatable, user-friendly, suitable for different types of studies, and applicable across all sociodemographic levels.

**Methods:**

Two measures were developed in light of the literature and expert opinion and piloted for clarity and ease of use. Following this, four studies were undertaken across different cohorts (including a sample of students, both ‘Food preparation novices’ and ‘Experienced food preparers’, and a nationally representative sample) to assess temporal stability, psychometrics, internal consistency reliability and construct validity of both measures. Analysis included T-tests, Pearson’s correlations, factor analysis, and Cronbach’s alphas, with a significance level of 0.05.

**Results:**

Both measures were found to have a significant level of temporal stability (*P* < 0.001). Factor analysis revealed three factors with eigenvalues over 1, with two items in a third factor outside the two suggested measures. The internal consistency reliability for the cooking skills confidence measure ranged from 0.78 to 0.93 across all cohorts. The food skills confidence measure’s Cronbach’s alpha’s ranged from 0.85 to 0.94. The two measures also showed a high discriminate validity as there were significant differences (*P* < 0.05 for cooking skills confidence and *P* < 0.01 for food skills confidence) between Food preparation novices’ and ‘Experienced food preparers.’

**Conclusions:**

The cooking skills confidence measure and the food skills confidence measure have been shown to have a very satisfactory reliability, validity and are consistent over time. Their user-friendly applicability make both measures highly suitable for large scale cross-sectional, longitudinal and intervention studies to assess or monitor cooking and food skills levels and confidence.

## Background

The consumption of food prepared in the home environment has been associated with an improved diet quality and better weight control [1, 2]. As obesity is increasingly becoming a worldwide epidemic, any methods that can contribute to its reduction must be considered and all measures should be taken to help their implementation. The promotion and increase of home meal preparation is one strategy in a multidisciplinary approach to tackling this issue [3], however, a culinary transition has been reported within the literature [4]. This transition explores the possibility that individuals may not have the necessary skill level to prepare a meal [5, 6]. In addition, numerous other barriers have been proposed for home meal preparation and cooking from scratch including; lack of time (real and perceived), perceived affordability of healthy foods versus convenience products, longer working hours, a dislike for cooking or the effect of negative previous experiences, enjoying eating out and take-away foods, accessibility and considering it too much effort [7–10]. In a possible attempt to overcome this lack of skills or as a means to facilitate the consumption of food without cooking, the use of convenience and processed food has increased, thereby reducing the need for cooking skills. The increase in the use of these products may in fact contribute to the obesity epidemic as these products are typically high in fats and sugars [11–14]. In light of this, there is a need to assess the level of cooking skills within the population, and intervene where necessary.

Cooking skills have been defined as a set of physical or mechanical skills used in the production of a meal encompassing cooking methods (e.g. boiling) and food preparation techniques (e.g. peeling a vegetable), in addition to this they are also said to include conceptual and perceptual skills such as understanding the transformation food undergoes when heat is applied, i.e. knowing that chicken is fully cooked from its colour [15, 16]. Aside from the cooking skills needed to prepare a meal, there is a wider set of skills involved in the entirety of the meal preparation process known as Food skills [17, 18]. Food skills include the knowledge and skills to be able to select and prepare food with the available resources, to produce a nutritionally balanced, age appropriate and satisfying meals for those that are consuming it, this includes meal planning, shopping, budgeting, resourcefulness, and label reading [17, 19]. These skills are essential to prepare a meal in the home environment [17].

While some measures for cooking and food skills currently exist, they have some limitations. A key issue is that food skills tend to be encompassed in existing measures and not considered as a stand-alone set of skills and therefore all necessary elements may not be included [20]. Other limitations of previous measures include: the specific mention of certain foods which may not be transferable to all cultures, developed for specific intervention groups, and not having succinct definitions of the skills being measured [20, 21]. Some of the measures also had issues with their validations including using mainly female samples, small sample sizes, test-retest biased sample, self-selection bias and highly educated samples [21, 22].

In recognition of these limitations, the current research aimed to develop and validate two separate measures (one for cooking skills and one for food skills) that are clearly described, relatable, user-friendly, suitable for different types of studies, and applicable across all sociodemographic levels.

## Methods

### Tool construction

A review of cooking related literature was undertaken prior to tool development. This focused on the relationships between cooking and food skills and their impact on the healthiness of diets (for more details see [23]). The results of the critical appraisal of these articles in combination with the results from Safefood’s publication regarding food skills [24] underpinned the development of the measures. In addition, four semi-structured qualitative interviews with experts working in the area of health promotion including cooking and food skills interventions and education were conducted to validate the literature findings and to investigate whether anything missing from the literature that needed to be included. All experts had an undergraduate degree in Home Economics with either a masters or PhD in Home Economics, Nutrition, Food Science or related discipline. The interviews questioned areas such as diet quality influencers, the role of cooking and food skills on diet and their perceptions on what encompasses cooking and food skills. A template analysis (a form of thematic analysis used in qualitative research, emphasising the use of hierarchical coding and the development of a coding template that is continuously refined [25]) was used to assess the relevant information for the measures and the overall survey, including the need for clear terminology.

Two measures were developed - the cooking skills confidence measure, consisting of 14 items; and the food skills confidence measure, consisting of 19 items (see Table 1 for items and corresponding sources). The two measures were tested using two different presentation methods: Computer Assisted Personal Interviewing (CAPI) and the traditional paper and pen (P/P) styled format. For the CAPI method, the question: *“Please tell us which of the following you do (or use):”* is asked. The skills that are used are recorded. Following this, the question *“On a scale from 1 to 7 where 1 means very poor and 7 means very good, please say how good you are at…* [substitute each one they stated they used in previous question],” and only the skills that were previously stated as in use are rated and this creates the confidence measure. The confidence score is the sum of the 1 to 7 ratings for the skills that were stated as used. This process is repeated for both the cooking skills and food skills measures. If a skill is not used, it is scored a zero for that skill. There is also no classification of the confidence measures. For the paper and pen presentation, the participant is asked to *“say how good you are at each task on a scale of 1-7, where 1 is very poor and 7 very good,”* with a *‘Never/rarely do it’* option also given. The scoring of the measure is the same as the CAPI, where the confidence measure is a sum of the ratings of the items and a zero if a participant ticked that they ‘Never/rarely did it.’

**Table 1:** Cooking skills and food skills items

**Cooking skills** ^**a**^
Cooking Method
1. ‘Chop, mix and stir foods, for example chopping vegetables, dicing an onion, cubing meat, mixing and stirring food together in a pot/bowl’
2. ‘Blend foods to make them smooth, like soups or sauces’ (using a whisk/blender/food processor etc.)
3. Steam food (where the food doesn’t touch the water but gets cooked by the steam)
4. Boil or simmer food (cooking it in a pan of hot, boiling/bubbling water)
5. Stew food (cooking it for a long time (usually more than an hour) in a liquid or sauce at a medium heat, not boiling) e.g. beef stew
6. Roast food in the oven, for example raw meat/chicken, fish, vegetables etc.
7. Fry/stir-fry food in a frying pan/wok with oil or fat using the hob/gas rings/hot plates
8. Microwave food (not drinks/liquid) including heating ready-meals
Food Preparation Techniques
9. Bake goods such as cakes, buns, cupcakes, scones, bread etc., using basic/raw ingredients or mixes
10. Peel and chop vegetables (including potatoes, carrots, onions, broccoli)
11. Prepare and cook raw meat/poultry
12. Prepare and cook raw fish
13. Make sauces and gravy from scratch (no ready-made jars, pastes or granules)
14. Use herbs and spices to flavour dishes
**Food skills** ^**b**^
Meal Planning and Preparing
1…plan meals ahead? (e.g. for the day/week ahead)
2…prepare meals in advance? e.g. packed lunch, partly preparing a meal in advance
3…follow recipes when cooking?
Shopping
4…shop with a grocery list?
5…shop with specific meals in mind?
6…plan how much food to buy?
Budgeting
7…compare prices before you buy food?
8…know what budget you have to spend on food?
9…buy food in season to save money?
10…buy cheaper cuts of meat to save money?
Resourcefulness
11…cook more or double recipes which can be used for another meal?
12…prepare or cook a healthy meal with only few ingredients on hand?
13…prepare or cook a meal with limited time?
14…use leftovers to create another meal?
15… keep basic items in your cupboard for putting meals together? e.g. herbs/spices, dried/tinned goods?
Label reading/consumer awareness
16…read the best-before date on food?
17…read the storage and use-by information on food packets?
18…read the nutrition information on food labels?
19…balance meals based on nutrition advice on what is healthy?

Source:

^a^Devised from National Diet and Nutrition Survey (NDNS) Year 1 [35], Barton et al. [20], Condrasky et al. [21], Chun & Worsley [36] and by the research team.

Participants asked to rate how good they are at each skill, on a scale of 1–7, where 1 is very poor and 7 is very good. If a skill is not used, an option of ‘never/rarely do it’ is available for participants to tick.

^b^Devised from National Diet and Nutrition Survey (NDNS) Year 1 cooking items [35], Barton et al. [20], Condrasky et al. [21], and by the research team

Participants asked to rate how good they are at each skill, on a scale of 1–7, where 1 is very poor and 7 is very good. If a skill is not used, an option of ‘never/rarely do it’ is available for participants to tick

The data for the present research was collected as part of the *‘Impact of cooking and related food skills on the healthiness of diets’* project, a research project carried out on the island of Ireland (IOI) and funded by Safefood Ireland, The Food Safety Promotion Board. Ethical approval for this research was received from Queen’s University Belfast Research Ethics Committee and each study was conducted in line with the guidance given in the Declaration of Helsinki. Participants consented to partake in the research and were aware they could remove themselves at any point. The measures were included in a larger survey assessing diet quality, psychological components, and demographic details (further information on other elements assessed in the overall survey can be seen in Lavelle et al. [26] and McGowan et al. [27]). Approximately 40 internal pilot surveys (convenience sampling of contacts of the research team across the collaborating universities on the IOI) took place; and 14 pilot survey field based interviews took place with SMR (data collection sub-contractors) during the refinement of the survey tool. These pilots assessed the clarity of the questions and how easy participants found the measures to complete and resulted in minor amendments to the wording of questions in the survey. The pilot data collected was not used in the statistical validation of the measures, however, readability and usability was assessed in these pilots. At this piloting phase, after minor amendments to language, participants at the various stages of piloting, verbally reported that they found the measures clear in meaning and not time consuming. After piloting, there were four studies which together assed the reliability and validity of the two measures. As missing data was scattered randomly through the datasets (assessed by creating dummy variables and running independent t-tests), missing data was handled using listwise deletion [28]. All data were analysed using SPSS version 22 (IBM Corporation, 2013) and a significance level was set at 0.05 for all analysis.

### Study 1

Study 1 was conducted to assess whether the skills were appropriately classified into ‘cooking skills’ and ‘food skills,’ as the skills included in the measures were derived both from previous literature and expert opinion. In addition, this study investigated the convergent validity of the measures, to assess whether the two measures of cooking skills and food skills are related but not a single factor, as they theoretically should be related yet distinct.

A nationally representative sample of 1049 adults between the ages of 20–60 years, responsible for preparing a main meal at least once per week was collected. All sampling was completed by SMR, a market research company (in line with their sampling procedures). An overview of demographic characteristics can be seen in Table 2. Further details on sampling and procedure can be found in previous publications [26, 27]. Briefly, the measures were completed in this sample as part of the larger survey by Computer Assisted Personal Interviewing and were conducted by fully trained interviewers in participants’ homes between October and December 2014. In total, 1172 potential participants were approached to partake in the survey. One hundred and twenty-three participants did not participate in the survey (this represents 10.50% of potential participants approached) due to ineligibility or for other reported reasons including no interest, no time, did not feel comfortable having the interviewer in their home, etc.

**Table 2:** Sociodemographic characteristics of nationally representative sample in Study 1

Characteristic	M	SD
*Age*	39.72	11.84
	*N*	%
*Jurisdiction of Residence*		
Northern Ireland	312	29.7
Republic of Ireland	737	70.3
*Gender*		
Female	590	56.2
Male	459	43.8
*Level of Education*		
None	3	0.3
Primary School	11	1.0
Secondary School (Junior Cert/GCSE – age 15/16)	121	11.5
Secondary School (Leaving Cert/A Level – age 17/18)	351	33.5
Additional Training (e.g. NVQ, BTEC, FETAC, FAS)	305	29.1
University Undergraduate	153	14.6
University Postgraduate	105	10.0
*Perceived Weight Status*		
Very underweight	5	0.5
Slightly underweight	58	5.5
About the right weight	601	57.3
Slightly overweight	334	31.8
Very overweight	51	4.9

Results from this sample were used to conduct factor analysis using oblique rotation (direct oblimin) to assess the item classification into the two measures, interpreting both the Pattern Matrix and the Structure Matrix. Correlations were used for the investigation of the convergent validity. In addition, the internal consistency reliability of the measures was tested using Cronbach’s alpha.

### Study 2

This study measured the test-retest reliability of the measures to assess their temporal stability. This sample was also used to examine the internal consistency reliability of the measures in the P/P format. Study 2 consisted of a sample of 23 ‘Food preparation novices’ students from Ulster University, recruited by the research team. Students were classified as ‘Food preparation novices’ if they were enrolled on a course that consisted of no nutrition, hospitality, food marketing or food product and innovation orientated modules. The participants were made aware that they could withdraw at any time and that their details would remain confidential. The students were aged 18–27. Their demographic characteristics can be seen in Table 3. These participants completed a pen and paper version of the two measures and two weeks later completed the measures again. Of the students recruited; 56.5% (13 students) completed both measures at both time points, 30.4% (7 students) had partial completion of both measures at both time points (some element was missing, for example a section of one of the measures) and 13% (3 students) completed both measures at only one time point. Mean differences and Pearson correlations were used to verify the consistency of the results over time. Again, Cronbach’s Alpha’s were used to assess the internal consistency reliability of the P/P measures.

**Table 3:** Sociodemographic characteristics of student sample in Study 2

Characteristic	M	SD
*Age*	19.22	1.91
	N	%
*Jurisdiction of Residence*		
Northern Ireland	23	100
*Gender*		
Female	17	73.90
Male	6	26.10
*Level of Education*		
Secondary School (Leaving Cert/A Level – age 17/18)	19	82.6
Additional Training (e.g. NVQ, BTEC, FETAC, FAS)	2	8.7
University Undergraduate	2	8.7
*Perceived Weight Status*		
Slightly underweight	1	4.3
About the right weight	15	65.2
Slightly overweight	5	21.7
Very overweight	1	4.3

### Study 3

Study 3 was used to test the discriminate validity of the measures between those with high levels of cooking and food skills, and those with low levels. In addition, this sample was used to further assess the internal consistency reliability of the P/P measures. This study consisted of a sample of 57 students. This sample completed a modified version of the larger survey (some questions regarding dietary intake, sources of learning etc. were removed to reduce the time needed for completion) as the overall aim was to test the discriminant validity of the two measures.

The student sample consisted of students from the Ulster University, Northern Ireland and St. Angela’s College Sligo, Ireland, recruited by the researchers. All students were made aware that they could withdraw at any time and that their confidentiality would be kept intact. The students were either studying a Business-related degree or were studying Home Economics in their second year and were classified as ‘Food preparation novices’ (same classification as Study 2) and ‘Experienced food preparers’ (each year required to complete a minimum of 80 h of practical cooking externally to any completed within class time, in addition to studying food science and nutrition). The ‘Experienced food preparers’ sample were aged 18–26 and the ‘Food preparation novices’ were aged 19–24. Their characteristics can be seen in Table 4. This sample also completed a pen and paper version of the survey.

**Table 4:** Sociodemographic Characteristics of Student sample in Study 3

Characteristic	M	SD
Experienced Food Preparers		
*Age*	19.70	1.27
	N	%
*Jurisdiction of Residence*		
Republic of Ireland	40	100
*Gender*		
Female	39	97.5
Male	1	2.5
*Level of Education*		
Secondary School (Leaving Cert/A Level – age 17/18)	34	85.0
Additional Training (e.g. NVQ, BTEC, FETAC, FAS)	1	2.5
University Undergraduate	4	10.0
University Postgraduate	1	2.5
*Perceived Weight Status*		
About the right weight	19	47.5
Slightly overweight	16	40.0
Very overweight	2	5.0
Characteristic	M	SD
Food preparation novices		
*Age*	21.35	1.41
	N	%
*Jurisdiction of Residence*		
Northern Ireland	17	100
*Gender*		
Female	11	64.7
Male	6	35.3
*Level of Education*		
Secondary School (Leaving Cert/A Level – age 17/18)	4	23.0
Additional Training (e.g. NVQ, BTEC, FETAC, FAS)	1	6.0
University Undergraduate	11	65.0
*Perceived Weight Status*		
Slightly underweight	1	5.9
About the right weight	6	35.3
Slightly overweight	10	58.8

The discriminate validity was tested between the ‘Experienced food preparers’ and the ‘Food preparation novices’ students using T-tests. Internal consistency reliability was investigated using Cronbach’s Alpha.

### Study 4

Study 4 was conducted to assess differences between the CAPI and the P/P method in relation to the confidence scores of the measures. This study sample consisted of a combination of the samples in study 2 and study 3 (keeping the Home Economics students as a separate group as they have greater skills and this could inflate the scores and introduce bias), representing the P/P method, and due to sample size differences, a randomly selected 38 participants from the IOI sample in Study 1, representing the CAPI method. Here three groups were compared two P/P method groups (Home Economic students and a combination of the non-Home Economics students from studies 2 and 3) and one CAPI method group (random selection from the IOI cohort). As the scoring is the same for both methods, one-way ANOVAs were conducted with Bonferroni post hoc analysis, to investigate differences in the cooking skills confidence measure and the food skills confidence measure between the two methods of presentation.

## Results

### Psychometrics and internal consistency reliability

Participants’ cooking and food skills confidence were measured using the following 14 items and 19 items respectively in Table 5 and their mean scores on each item can be seen. For the psychometric testing, three factors with eigenvalues over 1 were found - one main component accounts for 65.26% variance, then the second one 8.2% of the variance and component 3 just 3.2% of the variance. All factor loadings were above the minimum criterion. The pattern matrix indicated the original cooking skills and food skills measures with some minor exceptions (see Table 6). The items falling into this third component consisted of the items: ‘*Bake goods such as cakes, buns, cupcakes, scones, bread etc., using basic/raw ingredients or mixes’* and *‘Microwave food (not drinks/liquid) including heating ready-meals.’* In the Structure Matrix both the microwaving (Factor 1 = 0.656, Factor 2 = 0.557, Factor 3 = −0.537) and Baking (Factor 1 = 0.677, Factor 2 = 0.477) had highest loading in Factor 1 (Cooking skills). Four ‘food skill’ items had higher loadings in Factor 1 (Cooking Skills); Buying in Season, using leftovers to create another meal, Keeping Basics in the cupboard and Reading the best before date. In the Structure Matrix, Buying in Season had higher loading onto Factor 2 (Factor 1 = 0.758, Factor 2 = 0.764) and using leftovers to create another meal had the same loading on both Factors (Factor 1 and 2 = 0.798). Keeping basics in a cupboard and reading the best before date had higher loadings on Factor 1 in the Structure Matrix (Factor 1 = 0.789, Factor 2 = 0.731 and Factor 1 = 0.793 and Factor 2 = 0.758, respectively). The internal consistency reliability measured by Cronbach’s Alpha for the cooking skills confidence measure ranged from 0.78 (Study 3 Food preparation novices/Experienced food preparer students) to 0.93 (Study 1 IOI cohort), please see Table 7 for each cohorts Cronbach alpha. The food skills confidence measure’s Cronbach’s alpha’s ranged from 0.89 (Study 2 student cohort) to 0.94 (Study 1 IOI cohort) (see Table 7).

**Table 5:** Cooking skills and food skills measures use, mean and standard deviations

**Cohort**	**Study 1: Nationally Representative (CAPI)**				**Study 2: Students (P/P)**				**Study 3: Food Preparation Novices (P/P)**				**Study 3: Experienced Food Preparers (P/P)**			
**N**	1049				23				17				40			
**Cooking Skills**	**Usage**		**Confidence** **(rated 1–7)**		**Usage**		**Confidence (rated 1–7)**		**Usage**		**Confidence (rated 1–7)**		**Usage**		**Confidence (rated 1–7)**	
	***N***	**%**	**Mean**	**SD**	***N***	**%**	**Mean**	**SD**	***N***	**%**	**Mean**	**SD**	***N***	**%**	**Mean**	**SD**
1. Chop, mix and stir foods	401	38.2	5.88	1.112	22	95.7	5.65	1.70	17	100	5.88	1.22	40	100	6.65	0.70
2. Blend foods to make them smooth, like soups or sauces	140	13.3	6.01	1.158	17	73.9	3.70	2.67	14	82.4	4.50	2.25	40	100	6.45	1.04
3. Steam food	186	17.7	5.90	1.153	17	73.9	2.61	2.27	16	94.1	3.88	2.06	34	85.0	4.62	2.26
4. Boil or simmer food	427	40.7	5.75	1.141	22	95.7	5.91	1.54	17	100	5.47	1.28	40	100	6.47	0.75
5. Stew food	369	35.2	5.91	1.074	15	65.2	2.65	2.48	16	94.1	4.76	1.86	33	82.5	4.05	2.33
6. Roast food in the oven	404	38.5	5.86	1.089	20	87.0	4.57	2.39	17	100	5.18	1.51	40	100	6.35	0.83
7. Fry/stir-fry food in a frying pan/wok with oil or fat	407	38.8	5.71	1.169	21	91.3	5.26	2.12	17	100	6.35	1.00	40	100	6.48	0.91
8. Microwave food	189	18.0	5.98	1.266	22	95.7	5.87	1.79	14	82.4	4.82	2.72	34	85.0	4.95	2.53
9. Bake goods	110	10.5	5.55	1.310	19	82.6	3.96	2.40	14	82.4	3.88	2.06	39	97.5	6.40	1.41
10. Peel and chop vegetables	479	45.7	5.81	1.085	21	91.3	5.43	2.23	16	94.1	6.00	1.00	40	100	6.80	0.46
11. Prepare and cook raw meat/poultry	517	49.3	5.69	1.136	19	82.6	4.61	2.59	15	88.2	5.56	1.71	39	97.5	6.48	1.20
12. Prepare and cook raw fish	231	22.0	5.69	1.231	10	43.5	2.00	2.65	13	76.5	4.13	2.63	34	85.0	4.79	2.26
13. Make sauces and gravy from scratch	153	14.6	5.75	1.256	11	47.8	2.17	2.67	14	82.4	3.94	2.29	33	82.5	3.45	2.04
14. Use herbs and spices	273	26.0	5.71	1.173	21	91.3	4.48	2.21	15	88.2	5.50	2.16	39	97.5	5.83	1.62
**Overall Cooking skills scores**			47.78	29.32			58.83	16.45			71.07	15.32			79.67	10.42
**Cohort**	**Study 1: Nationally Representative (CAPI)**				**Study 2: Students (P/P)**				**Study 3: Food Preparation Novices (P/P)**				**Study 3: Experienced Food Preparers (P/P)**			
**N**	1049				21				17				40			
	**Usage**		**Confidence**		**Usage**		**Confidence**		**Usage**		**Confidence**		**Usage**		**Confidence**	
**Food Skills**	***N***	**%**	**Mean**	**SD**	***N***	**%**	**Mean**	**SD**	***N***	**%**	**Mean**	**SD**	***N***	**%**	**Mean**	**SD**
1.Plan meals ahead?	283	27.0	5.83	1.138	20	95.2	4.29	1.93	12	70.6	3.47	2.72	39	97.5	5.44	1.48
2…prepare meals in advance?	183	17.4	5.82	1.242	18	85.7	3.52	2.18	12	70.6	3.35	2.55	36	90.0	4.95	1.93
3…follow recipes when cooking?	237	22.6	5.77	1.233	21	100	4.71	1.90	12	70.6	3.88	2.76	36	90.0	6.05	1.53
4…shop with a grocery list?	299	28.5	5.94	1.221	19	90.5	4.48	2.29	13	76.5	3.76	2.59	37	92.5	5.46	1.85
5…shop with specific meals in mind?	481	45.9	5.80	1.103	19	90.5	4.14	2.27	15	88.2	4.35	2.37	39	97.5	6.05	1.15
6…plan how much food to buy?	326	31.1	5.78	1.159	19	90.5	4.38	2.42	14	82.4	3.76	2.51	39	97.5	5.95	1.19
7…compare prices before you buy food?	238	22.7	5.82	1.230	17	81.0	4.14	2.69	15	88.2	5.24	2.56	38	95.0	5.21	1.87
8…know what budget you have to spend on food?	248	23.6	6.03	1.112	18	85.7	4.43	2.80	15	88.2	5.12	2.45	37	92.5	5.47	1.64
9…buy food in season to save money?	150	14.3	5.89	1.191	13	61.9	2.29	2.24	13	76.5	2.65	1.97	38	95.0	4.62	1.79
10…buy cheaper cuts of meat to save money?	101	9.6	5.82	1.328	12	57.1	2.38	2.31	10	58.8	2.59	2.55	37	92.5	4.73	1.96
11…cook more or double recipes which can be used for another meal?	139	13.3	5.94	1.205	15	71.4	2.81	2.44	15	88.2	3.53	2.15	38	95.0	5.54	1.60
12…prepare or cook a healthy meal with only few ingredients on hand?	313	29.8	5.80	1.100	18	85.7	3.57	2.38	16	94.1	4.47	1.77	39	97.5	5.20	1.51
13…prepare or cook a meal with limited time?	300	28.6	5.83	1.087	19	90.5	4.24	2.05	15	88.2	5.00	2.00	40	100	5.95	1.26
14…use leftovers to create another meal?	303	28.9	5.79	1.205	18	85.7	3.76	2.49	16	94.1	3.88	2.06	39	97.5	5.25	1.79
15… keep basic items in your cupboard for putting meals together?	493	47.0	5.74	1.243	20	95.2	4.52	1.89	17	100	5.35	1.50	39	97.5	6.26	1.29
16…read the best-before date on food?	431	41.1	5.95	1.109	20	95.2	5.62	2.01	16	94.1	5.44	2.00	40	100	6.58	0.75
17…read the storage and use-by information on food packets?	280	26.7	5.90	1.158	16	76.2	4.10	2.86	17	100	5.24	2.17	39	97.5	5.95	1.52
18…read the nutrition information on food labels?	135	12.9	5.89	1.196	19	90.5	3.29	1.93	16	94.1	3.88	2.57	39	97.5	5.75	1.61
19…balance meals based on nutrition adviceon what is healthy?	119	11.3	6.06	1.033	18	85.7	2.62	1.91	15	88.2	3.53	2.43	39	97.5	5.37	1.66
**Overall food skills scores**			45.82	38.64			74.76	27.64			81.31	29.15			105.64	17.78

**Table 6:** Study 1: Summary of Exploratory Factor Analysis for cooking and food skills measures using direct oblimin oblique rotation and the pattern matrix (*N* = 1049)

	Factor Loadings		
Item	Factor 1: Cooking Skills	Factor 2: Food Skills	Factor 3: Other
1) Chopping, mixing, stirring	0.82		
2) Blending	0.80		
3) Steaming	0.90		
4) Boiling	0.95		
5) Stewing	0.89		
6) Roasting	0.84		
7) Frying/Stir-frying	0.86		
8) Microwaving	0.55		−0.48
9) Baking	0.61		0.46
10) Peeling	0.92		
11) Preparing/cooking raw meat/poultry	0.83		
12) Preparing/cooking raw fish	0.83		
13) Making sauces	0.88		
14) Using herbs/spices	0.82		
1) Planning ahead		0.74	
2) Preparing in advance		0.61	
3) Following recipes		0.76	
4) Grocery List		1.01	
5) Specific meals		0.76	
6) Planning how much to buy		0.78	
7) Comparing prices		1.07	
8) Knowing food budget		0.88	
9) Buying in season	0.46	0.43	
10) Buying cheaper cuts		0.89	
11) Doubling recipes		0.75	
12) Cooking healthy with a few ingredients		0.57	
13) Cooking with limited time	0.47	0.49	
14) Using leftovers	0.50	0.43	
15) Keeping basics in cupboard	0.56		
16) Reading best before	0.53		
17) Reading storage/use-by	0.42	0.45	
18) Reading nutrition information on labels		0.70	
19) Balancing meals based on nutrition advice		0.67	
Eigenvalues	21.54	2.72	1.07
% of Variance	65.26	8.23	3.24

*All factor loadings were greater than 0.162, the criterion for the sample size [37], the factor loadings are regression coefficients as they are from the pattern matrix [38]

**Table 7:** Internal Consistency reliability of the cooking skills and food skills confidence measures

Cohort	No. of items	Range	Mean Score^a^	SD	*n*	α
**Cooking Skills**						
Study 1: Island of Ireland (*N* = 1049, CAPI)	14	0–98	47.78	29.32	1049	.93
Study 2: Test/Re-test Students (*N* = 23, P/P)	14	27–89	58.83	16.45	23	.79
Study 3: Experienced/Novice food preparers (*N* = 57, P/P)	14	46–98	71.1	15.3	15	.78
**Food Skills**						
Study 1: Island of Ireland (*N* = 1049, CAPI)	19	0–133	45.82	38.64	1049	.94
Study 2: Test/Re-test Students (*N* = 23, P/P)	19	18–134	74.76	27.64	21	.89
Study 3: Experienced/Novice Food Preparers (*N* = 57, P/P)	19	32–127	81.3	29.2	16	.93

^a^NB the paper and pencil version meant that participants rated their ability on almost all CS and FS items. The national survey means are lower as participants were first asked to highlight the CS and FS they used and only rated their confidence of those items

### Temporal stability

The temporal stability of the measures was assessed using the test re-test approach in study 2. As there was a large number of missing cases in the food skills confidence measure test re-test scores, a dummy variable was created and showed there was no significant differences (*P* = .902) between those that reported on both time points and those that were missing data on food skills confidence on the initial time. The results show (see Table 8) that both measures have acceptable temporal stability as there were no significant differences between the two mean confidence scores from the first time (T1) and the two weeks repeated measure (T2). In addition, a further assessment of temporal stability was conducted using correlations between the cooking skills confidence measure and the food skills confidence measure scores at T1 and T2. A significant correlation was found between cooking skills measures at T1 and T2 (*r* = .815, *P* < 0.001) and food skills measures (*r* = .872, *P* < 0.001) again illustrating the consistency of the measures over time.

**Table 8:** Study 2 (P/P): Temporal Stability of both cooking and food skills confidence measures

Measure	N	Time point	Mean	SD	T	df	Sig
Cooking Skills Confidence	20	T1	58.25	17.26			
		T2	59.00	19.75	−0.292	19	0.774
Food Skills Confidence	13	T1	74.15	28.34			
		T2	71.77	27.06	0.610	12	0.553

### Construct validity

A strong positive correlation was found between cooking skills confidence and food skills confidence (*r* = 0.76, *p* < 0.001) which indicates that the measures are measuring highly related components. In addition, there was a significant difference between the mean scores on both the cooking and food skills confidence measures between ‘Food preparation novices’ and ‘Experienced food preparers’ from study 3. Table 5 shows that the ‘Experienced food preparers’ home economics students scored had consistently higher mean confidence scores on the individual items than the ‘Food preparation novices’ students, with the exception of 3 items (stewing, making sauces, and comparing prices before buying). ‘Experienced food preparers’ had significantly better cooking skills confidence (F = 4.63, *P* < 0.05) and food skills confidence (F = 7.95, *P* < 0.01) scores than ‘Food preparation novices’ (see Table 9). Therefore, the measures were found to have a high convergent validity as they were highly related. In addition, as the measures were found to discriminate between those with high and low skills they also had notable discriminant validity.

**Table 9:** Study 3 (P/P): Discriminant validity of both cooking and food skills confidence measures

Measure	Student Type	*N*	Mean	SD	F	Sig
Cooking Skills Confidence	Food Preparation Novices	15	71.07	15.32		
	Experienced Food Preparers	39	79.67	10.42	4.63	.036
Food Skills Confidence	Food Preparation Novices	16	81.31	29.15		
	Experienced Food Preparers	34	105.64	17.78	7.95	.007

### Differences between CAPI and P/P methods of presentation of measures

Results show that on both the cooking skills confidence and the food skills confidence measures there is a significant difference in scores between the two different methods (*P* < 0.005) (see Table 10). As seen in usage and confidence rating in Table 5 and discriminate validity in Table 9, Experienced Food preparers are consistently higher for their scoring, this difference is also seen in the comparison of the three groups in Table 10.

**Table 10:** Study 4: Differences between the different methods of presentation of the measures

	Range	F (df)	Significance	Non-Home Ec Students (P/P, *N* = 38)	Home Ec Students (P/P, *N* = 39)	Random Selection from IOI (CAPI, *N* = 38)
			*P*	M (SD)	M (SD)	M (SD)
**Cooking skills Confidence**	0–98	28.63 (2112)	.000	63.66 (16.93)^a^	79.67 (10.42)^b^	45.29 (28.38)^c^
				*N* = 37	*N* = 37	*N* = 38
**Food skills Confidence**	0–134	52.50 (2109)	.000	77.60 (28.10)^a^	106.16 (17.82)^b^	39.74 (35.52)^c^

Superscript letters depict where significant differences (P < 0.05) are found

#### Frequency of usage of skills

Table 5 shows an overview of the reported frequency of usage and mean confidence score of all items for each group and following is the top reported used skills in each group. For the study 1 cohort, Preparing and cooking raw meat/poultry was the top reported skill (49.3%), followed by Peel and chopping vegetables (45.7%), Boiling or simmering food (40.7%), Frying/Stir-frying (38.8%), and Roasting/Baking food in the oven (38.5%). The Study 2 sample reported using Microwaving, Chopping, and Boiling (95.7%) and Frying/stir-frying, Peeling and chopping vegetables/using herbs and spices (91.3%). In Study 3, the ‘Food preparation Novices’ reported using Chopping, Boiling, Roasting, and frying/stir-frying (100%). In comparison to the ‘Experienced Food Preparers’ who reported using Peeling, Frying/stir-frying, Roasting, Boiling, Blending and chopping (100%) and Baking, preparing and cooking raw meat/poultry, and using herbs and spices (97.5%). In addition, for this groups reported usages for any cooking skill was not below 82.5%.

For the food skills Study 1 reported usage of keeping basics in the cupboard (47.0%), shopping with specific meals in mind (45.9%), and reading the best before (41.1%) as the top three. Study 2’s sample stated their usage of following a recipe (100%), keeping basics in the cupboard, reading the best before, and planning ahead (all 95.2%). In Study 3, the ‘Food Preparation Novices’ reported keeping basics in the cupboard and reading the use-by (100%) and reading the nutrition information, reading the best before, using leftovers to create another meal, and creating a meal from few ingredients (94.1%). Comparatively, the ‘Experienced Food Preparers’ had no reported usage below 90%, all of the sample reported reading the best before and being able to create a meal with limited time. 97.5% of this sample reported the usage of Planning ahead, shopping with specific meals in mind, planning how much to buy, preparing a meal with few ingredients, creating a meal using leftovers, keeping basic ingredients in the cupboard, reading the use-by date, reading the nutrition information and balancing meals based on nutrition advice.

## Discussion

This paper describes the development and validation of two measurements to assess cooking skills confidence and food skills confidence. The measures were developed to include a full range of skills for both cooking (cooking method and food preparation techniques) and wider food skills (meal planning, shopping, budgeting, resourcefulness and label reading) through a literature review, expert interviews and piloting. The results indicate that both measures have substantial internal consistency reliability, construct and convergent validity and temporal stability, as well as being easy to complete and user-friendly measures.

Both measures were highly correlated with each other which showed that they are related but also psychometric testing indicated that they were two distinct measures. The psychometric testing showed that two items did not only fit into the two factors (cooking skills and food skills) but also into a third factor, however they were left in the cooking skills measure as the third factor consisted of only these two items and explained a small variance. In addition, in the Structure Matrix both these items had higher loadings in the Cooking skills Factor (Factor 1). Furthermore, as Nunnally and Bernstein [29] argue the intention of factor analysis is to reduce variables to ‘more substantive’ underlying factors and the use of a cut-off point of an eigenvalue of 1 for factor extraction (as recommended by Kaiser [30]) is over simplified and fundamentally flawed. Therefore, as recommended in Field [31], a scree plot was used to assess factors for extraction and only the initial two factors were to the left of the point of inflection. The two items that fell into the third factor were microwaving food and baking goods such as cakes. It is suggested that although microwaving may be used in meal preparation, for example microwaving a ready meal, it is not seen as a cooking skill and therefore participants did not rate this as they did not use it in cooking a meal. However, as a wide range of cooking abilities were recruited for, including those with very limited skill, participants had to be responsible for preparing a main meal at least once a week which could include heating a ready meal. By removing microwaving as a skill it may have excluded some participants that potentially use microwaving as their only skill or in combination with one or two others and therefore it was decided to keep microwaving as part of the scale. The other item that was questionable but that was kept was baking. Baking may be seen as different to cooking in itself, with its own separate skills, however, there is a certain amount of overlap and there are elements of baking that may be used in meal preparation including making fresh bread or making pastry for pies. Therefore, the item was included in the cooking skills confidence measure, however, future studies may consider developing and validating a baking skills confidence measure in itself. Additionally, the two items ‘Keeping basic ingredients in the cupboards,’ and ‘Reading the best before date’ had higher loadings on the Cooking Skills Factor. As the two measures are correlated, it is understandable that some items would load on both measures, however, as conceptually these items are not considered ‘cooking skills’ and have been identified as food skills in previous literature [17], they were left in the food skills measure and further testing of these items is needed.

The temporal stability of the measures, assessed using a test-retest approach, was found to be highly correlated with both measures having no differences in their mean scores between both time points and with the correlations between the time points for both measures being significant. This highlights that scores remain consistent over time in a non-biased sample as the sample consisted of ‘Food preparation novices’ that had no previous experience of the measures or cooking/food education. Previous research has had problems with test re-test samples being biased due to their involvement in the development and testing of the measures [21].

The internal consistency reliability of both measures was >0.70 in all cohorts. Cronbach’s alpha’s > 0.70 have been established as satisfactory for non-clinical measures [32, 33]. Therefore, both the cooking skills confidence measure and the food skills confidence measure can be seen as highly reliable and they are measuring coherent concepts.

The two measures also showed a high discriminate validity as there were significant differences (*P* < 0.05 for cooking skills confidence and *P* < 0.01 for food skills confidence) between ‘Experienced food preparers’ (Home Economics students), proposed as high skilled students, and ‘Food preparation novices’, proposed as lower skilled students. Therefore, these measures would be able to distinguish between higher and lower skilled individuals and could potentially be used for pre-screening for cooking interventions aimed at low skilled individuals. This supports the overall high construct validity of both measures [33].

A difference in both the cooking skills confidence measure and food skills confidence measure scores were seen between the different methods of presenting the questions to the participants. This is an important factor to consider when choosing the method of presenting the measures to participants in future studies, as those that complete a P/P version of the scale may report inflated scores. If possible it is recommended using a tablet/computer to replicate the system used in CAPI where participants are presented with all the skills and choose which they use and then after completing this, they are then asked to rate their confidence on only the skills they stated they used. When presented with the question of usage and rating confidence at the same time, participants were more likely to give a rating for a skill even if they did not use it. Therefore, if using the P/P version of the measures this inflation of scores must be taken into account. Further when studying change in cooking skills the method used must be the same. In addition, the representativeness of the measures may be considered to be accurate considering the reported usage. For the cooking skills items, the top reported cooking skills may be considered reflective of the eating patterns of that group, the Study 1 group top skills could be considered to correspond with a traditional diet on the IOI, meat and vegetables, roasts, casseroles (that are still reported as the most common main meals on the IOI – data not reported here). The student samples report chopping, frying/stir-fry, boiling, peeling, microwaving, roasting in the oven, these skills could be considered fundamental to the quick, easy meals associated with a student diet. In addition, the top reported food skills across the different cohorts show a number of consistencies, such as keeping basic ingredients in cupboards and reading best before dates. This shows learned behaviours across the different groups or perhaps adherence to consistent health messages (checking the best before date).

Additionally, some interesting differences between the ‘Food preparation novices’ and the ‘Experienced food preparers’ can be seen. Experienced food preparers tended to use a higher number of skills more frequently than the food preparation novices. The high frequency use of skills such as peeling, blending and preparing or cooking raw meat/poultry may indicate a greater use of basic ingredients in this group. Future interventions should focus on increasing the number of skills used by lower skilled individuals to enable the use of more basic ingredients. Additionally, future research could investigate correlations between the use of different skills and the type of ingredients used in meal preparation. Furthermore, differences can be seen between usage of food skills among these groups. Experienced food preparers had a greater use of preparing a meal with limited time, as time has been identified as key barrier to home meal preparation [8], future research should investigate whether their use of more skills more frequently or that they have a greater confidence in a range of skills enables meal preparation in a limited time.

### Strengths and limitations

The new measures were developed in light of the literature and expert opinion and were found to be highly reliable and valid. In addition, the items do not revolve around specific foods, which increases the generalisability of the measures outside UK and Irish populations, although further studies to assess the validity of these measures in other populations are needed. In addition, although there were a large number of male respondents completing the measures, the majority were female. This may be reflective that women remain responsible for the meal preparation of most households [34]. However, this must be taken into consideration when generalising the results to men. A limitation of the test re-test study was the format in which the measures were presented, the question was split over two pages and some participants did not turn the page to complete the second half of the measure and therefore those measures could not be included in the analysis resulting in the lower response rate for the food skills confidence measure. Due to different answering formats, P/P versions of the measures were found to inflate the scores on both measures. Thus, interviewer led or computer based applications (where participants are only provided the opportunity to rate the skills they said they use) may provide the most accurate scores for these measures. However, to use as part of a paper based large scale cross-sectional survey, longitudinal research or as screeners/measures for interventions, rearranging the question to put “if you don’t do a skill” tick ‘Never/rarely do it’ first followed by confidence rating may help to make this version similar to the CAPI version.

## Conclusions

The developed cooking skills confidence measure and the food skills confidence measure have been shown to have a very satisfactory internal consistency reliability and validity. In addition, they have been shown to remain consistent over time. They are able to accurately distinguish between high and low skilled individuals and are user-friendly. While the choice of presentation of the measures must be considered, both measures are highly suitable for large scale cross-sectional research, longitudinal studies and interventions alike to assess or monitor cooking and food skills levels and confidence.

## Abbreviations

CAPI: Computer Assisted Personal Interviewing
IOI: Island of Ireland
P/P: Paper Pen
UK: United Kingdom
